# A novel evaluation system to monitor bone formation and β-tricalcium phosphate resorption in opening wedge high tibial osteotomy

**DOI:** 10.1007/s00167-014-2870-3

**Published:** 2014-02-05

**Authors:** T. Tanaka, Y. Kumagae, M. Chazono, S. Kitasato, A. Kakuta, K. Marumo

**Affiliations:** 1 Department of Orthopaedic Surgery, NHO Utsunomiya National Hospital, 2160 Shimo-Okamoto, Utsunomiya, Tochigi 329-1193 Japan; 2 Department of Orthopaedic Surgery, Jikei University School of Medicine, Tokyo, 105-8461 Japan

**Keywords:** β-TCP, Opening wedge HTO, Osirix, Bone formation

## Abstract

**Purpose:**

The aim of this study was to establish an evaluation system to monitor bone formation and beta-tricalcium phosphate (TCP) resorption in opening high tibial osteotomy (HTO).

**Methods:**

From 2003 to 2005, opening HTO was performed in 36 patients using a Puddu plate and β-TCP blocks with 60 and 75 % porosity. Thirty-one patients were used for evaluation. All patients underwent CT examination at 2 weeks and 6 years. The CT image data were divided into three areas, and CT values of each area were analysed using the imaging software, Osirix.

**Results:**

CT image analysis at 2 weeks showed that the mean CT-attenuation values (in Hounsfield units) of the implanted area with β-TCP of 60 % porosity, the implanted area with β-TCP of 75 % porosity, and cancellous bone were, 1,694.0 ± 94.2, 1,010.9 ± 81.1, and 178.0 ± 45.1, respectively. Six years after surgery, these values were 574.1 ± 273.5, 168.8 ± 75.1, and 174.9 ± 69.3, respectively.

**Conclusion:**

β-TCP with 75 % porosity was completely resorbed and replaced by bone. β-TCP with 60 % porosity was resorbed, but approximately 1/3 still remained even 6 years after surgery. The imaging software, Osirix, enabled scanning of the whole area to measure CT values. This system is the first to quantitatively evaluate β-TCP resorption and bone formation in opening HTO.

**Level of evidence:**

Laboratory studies.

## Introduction

Lateral closed wedge high tibial osteotomy (HTO) described by Jackson and Waugh [7] and Coventry [3] is an established technique to treat osteoarthritis of the medial compartment and varus deformity. This technique has gradually given way to medial opening wedge osteotomy. The advantages of a medial opening wedge osteotomy compared with a lateral closing wedge osteotomy include easier technique, achievement of more predictable correction [5], maintenance of bone stock, correction of the deformity close to its origin, and avoidance of the peroneal nerve, and, proximal tibiofibular joint. However, a suitable material to fill the opened defect in the medial opening procedure has not been identified. Although autogenous bone is the preferred graft material for filling bone defects [21], autogenous bone grafting has the disadvantages of co-morbidity at the donor site. Allografts have commonly been used as substitutes for autogenous bone grafts; significant problems associated with allografts include a low bone fusion rate and disease transmission [2, 8]. Recently, bone substitute materials have been advocated as alternatives to autografts and allografts. Hydroxyapatite is widely used as a bone substitute because of its excellent biocompatibility and osteoconductive properties [9, 14]. Although it biodegrades slowly and there is no progressive bone formation during the bone repair [6]. Koshino et al. [10] reported good long-term results after opening HTO using hydroxyapatite as a bone filler. If, however, severe varus or valgus knee deformities were to occur, fixing a component in the tibia during total knee arthroplasty might be difficult. In contrast, most implanted porous β-tricalcium phosphate (TCP) can be resorbed within a few years [17]. In previous studies, we used β-TCP with 75 % porosity, but this has a compressive strength of only 3 MPa, which is inadequate for weight-bearing sites until bone incorporation occurs. Thus, we recently developed a wedge-shaped β-TCP block with 60 % porosity for opening HTO. This β-TCP has a compressive strength of 22 MPa, which is approximately sevenfold greater than β-TCP with 75 % porosity. We previously reported the clinical results of bone formation and resorption of β-TCP using β-TCP blocks with 75 and 60 % porosity at 33 months [18]. During opening HTO, the opened defect was fixed with a Puddu plate, after which β-TCP with 75 % porosity was used to fill the cancellous bone defect. On the medial cortical bone side, however, a wedge-shaped β-TCP block with 60 % porosity was implanted in front of and at the back of the plate. The use of a β-TCP block with 60 % porosity avoided autogenous bone grafting and shortened the surgical time. The decreased porosity reduced pore interconnection, suggesting a longer period for β-TCP resorption. In that study, radiographic examination showed that β-TCP with 75 % porosity was almost completely resorbed, but some β-TCP with 60 % porosity remained. Other reports also evaluated β-TCP resorption in opening HTO using plain X-rays [1, 4, 11, 16, 19]. To our knowledge, no radiological rating system to monitor remodelling of β-TCP using CT images has been reported. The aim of this study was to establish a novel evaluation system to monitor bone formation and β-TCP resorption in opening HTO.

## Materials and methods

### Preparation of β-TCP

The β-TCP block used in this study was highly pure and provided by Olympus Biomaterials Co. Ltd., Tokyo, Japan. β-TCP was synthesized using a mechanochemical method (wet milling). Briefly, CaHPO_4_/H_2_O and CaCO_3_ at a molar ratio of 2:1 were mixed into a slurry with pure water and particles of zirconium in a pot mill for 24 h and dried at 80 °C. Calcium-deficient hydroxyapatite was converted to β-TCP by calcination at 750 °C for 1 h. After sintering of the β-TCP powder at 1,050 °C for 1 h, a porous β-TCP block with a mean pore size of 200 μm and a porosity of 75 % was obtained. A bimodal pore size distribution in the β-TCP block with 75 % porosity was observed, where one peak existed in a region of >100 μm and the other peak existed in a region of <5 μm. The β-TCP block with 60 % porosity was synthesized by the same method as the 75 % material, except that the amount of forming agent was changed. The mean compressive strength of β-TCP blocks with 60 and 75 % porosity was 22 and 3 MPa, respectively.

During opening HTO, the opened defect was fixed with a Puddu plate after which the cancellous bone defect was filled with 4 wedge-shaped β-TCP blocks of 75 % porosity. On the medial side, however, 3 wedge-shaped β-TCP blocks with 60 % porosity were implanted in front of and at the back of the plate. Eighteen patients received an opening of 10 mm and 13 patients had an opening of 12.5 mm. The volume of each piece of β-TCP block with 60 % porosity for a 10 mm opening was 1.06 cm^3^ and for a 12.5 mm opening was 1.39 cm^3^. Three pieces of β-TCP were implanted in all cases, but 1/3 of one piece was removed in the case of small knees. To fill the cancellous bone defects, 6–7 and 7–8 cm^3^ of β-TCP blocks with 75 % porosity were used for 10 and 12.5 mm openings, respectively.

Between 2003 and 2005, opening wedge HTO was performed on 36 patients using a Puddu plate and β-TCP with 60 and 75 % porosity. Thirty-one patients (22 women and 9 men) with a mean age of 64 years (range 51–77 years) at the time of surgery, were evaluated with a mean follow-up point of 75 months (range 72–78 months).

**Fig. 1:**
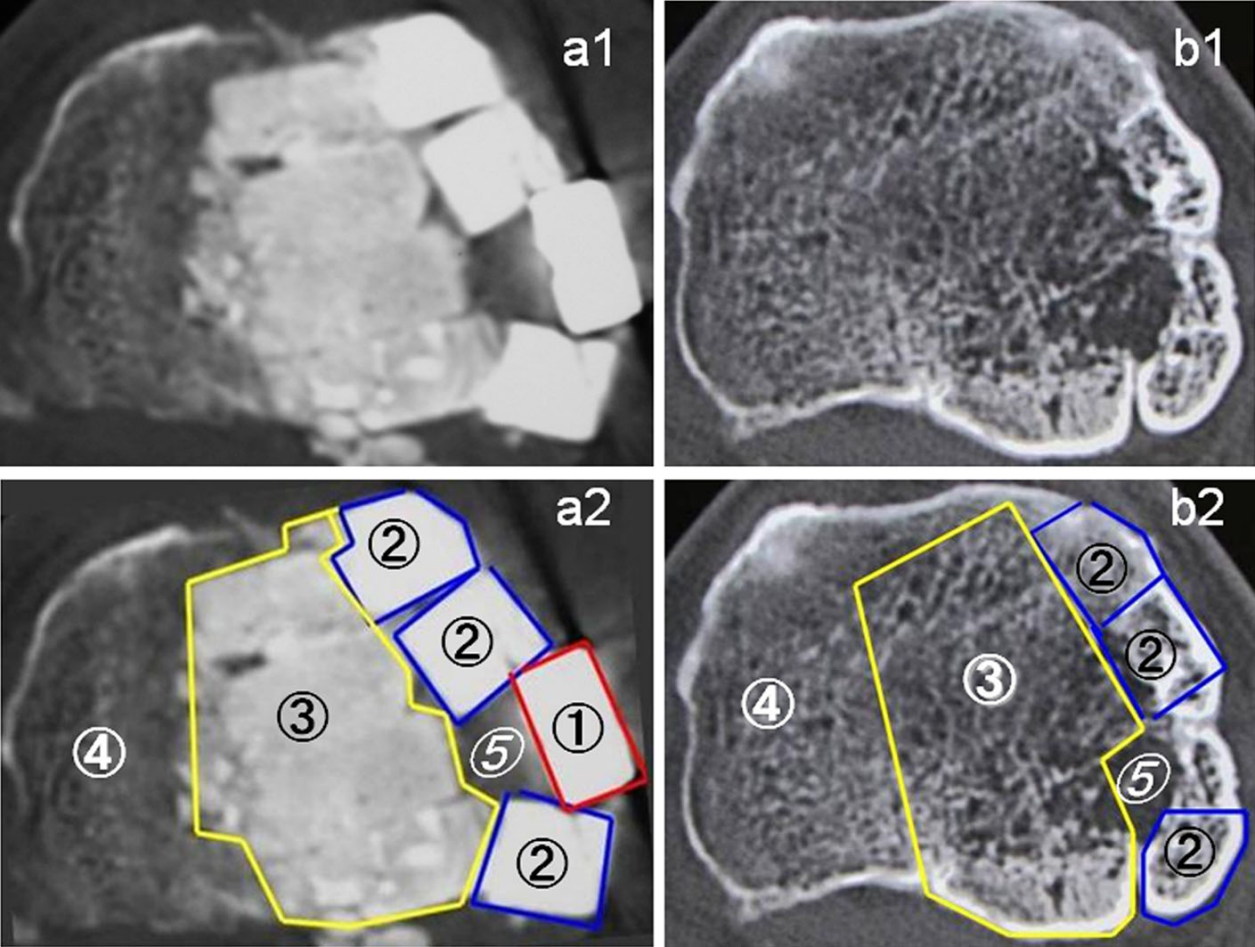
CT images of a 64-year-old man showing the centre of the osteotomy plane at 2 weeks (**a1**, **a2**) and 6 years (**b1**, **b2**). The mean CT values (HU) of the area implanted with β-TCP of 60 % porosity (②), the area implanted with β-TCP of 75 % porosity (③), hinged-cancellous bone (④), and the area with insufficient β-TCP implantation (*⑤*) were, 1,717.3, 1,044.8, 175.1, and 39.1, respectively. Six years after surgery, these values were 440.5, 162.0, 164.3, and 21.9, respectively. The area with insufficient β-TCP implantation (*⑤*) showed poor bone formation. ① indicates the area of the metal plate

Exclusion criteria in this study included (1) patients with standing lateral femorotibial angle of more than 184°; (2) patients who had an extension loss of more than 10°; (3) patients who had severe osteoarthritis in the patellofemoral joint; and (4) patients whose BMI was more than 27 (kg/m^2^). After surgery, the knee was protected with a hinged brace for 12 weeks. Partial weight-bearing was allowed at 3–4 weeks, and total weight-bearing was allowed at 5–6 weeks. All patients received follow-up evaluations at regular intervals in our outpatient clinic, and underwent radiographs and CT examinations at 2 weeks (Fig. 1a) and at 6 years (Fig. 1b). CT images parallel to the osteotomy plane were made and images at the centre were used to evaluate β-TCP resorption and bone formation. The CT image data were divided into 3 areas by tracing manually on the screen of the computer (Fig. 1-a2, b2). CT attenuation values [in Hounsfield units (HU)] of the area implanted with β-TCP of 60 % porosity, the area implanted with β-TCP of 75 % porosity, and hinged-cancellous bone were analysed using the imaging software, Osirix. Traces of the β-TCP blocks were still visible at 6 years post-operatively in some cases, but in most cases, it was hard to define the β-TCP implanted area. In those cases, CT images at 2 weeks were used to define the β-TCP implanted area. Circumferential cortical bone of the tibia was excluded from each value of β-TCP and cancellous bone.

### Reproducibility of the radiological evaluating system

The CT images were evaluated independently by two investigators (TT and YK). The two investigators re-evaluated the CT data one more time at 11 months after the first analysis. Inter-observer variability as well as intra-observer variability during the first and second rounds were determined using intraclass correlation coefficient, an appropriate summary statistic for determining the reliability of measurement [12, 20].

All patients provided informed consent prior to participation in this study, which was approved by NHO Utsunomiya National hospital ethical committee (ID 15-1).

### Statistical analysis

We used a paired *t* test to compare CT values of β-TCP implanted areas and cancellous bone.

A *P* value of less than 0.05 was regarded as statistically significant.

## Results

**Fig. 2:**
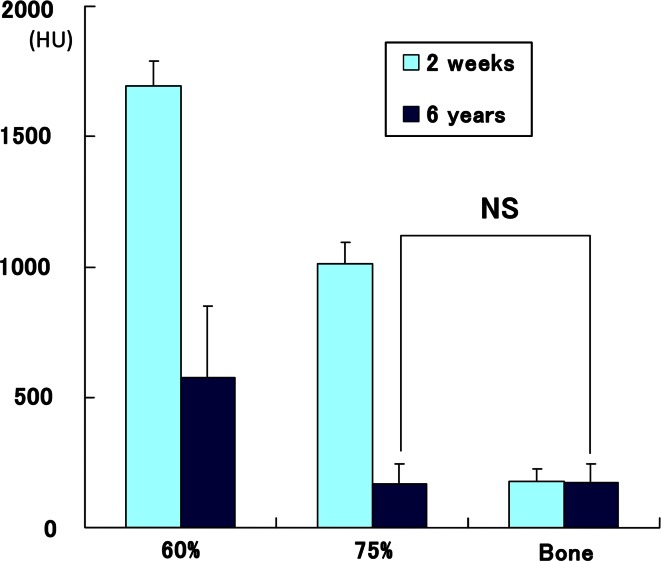
CT values (HU) obtained from each area of 31 cases at 2 weeks and 6 years. The CT values of β-TCP with 75 % porosity implanted area at 6 years were not statistically different from those of hinged-cancellous bone, indicating that β-TCP with 75 % porosity was completely resorbed and replaced by cancellous bone. The labels “60”, “75”, and “bone” indicate the area implanted with β-TCP of 60 % porosity, the area implanted with β-TCP of 75 % porosity, and hinged-cancellous bone, respectively

The mean preoperative standing lateral femorotibial angle was 181° (range 177°–184°), which was corrected to a mean angle of 169° post-operatively. No correction loss was observed at the final follow-up. Bone formation and β-TCP resorption were noted in all cases. CT image analysis containing the centre of the osteotomy plane showed that the mean CT value (HU) of the implanted area with β-TCP of 60 % porosity at 2 weeks and 6 years were 1,694.0 ± 94.2, and 574.1 ± 273.5, respectively. The mean CT value of the implanted area with β-TCP of 75 % porosity at 2 weeks and 6 years were 1,010.9 ± 81.1, and 168.8 ± 75.1, respectively. The mean CT value of cancellous bone at 2 weeks and 6 years were 178.0 ± 45.1, and 174.9 ± 69.3, respectively (Fig. 2). The values with the smallest standard deviation (SD) were for hinged-cancellous bone at 2 weeks and values with the largest SD were for areas implanted with β-TCP of 60 % porosity at 6 years.

Six years after surgery, there was no statistical difference of the CT values between the area implanted with β-TCP of 75 % porosity and cancellous bone, indicating that the β-TCP with 75 % porosity had been completely resorbed and replaced by bone. In contrast, the CT values indicated that approximately 1/3 of the β-TCP with 60 % porosity remained even 6 years after surgery (Fig. 2).

Significant difference of β-TCP resorption was not found in the two patients who smoked.

The intraclass correlation coefficients of the CT values of 60 % porosity β-TCP, 75 % porosity β-TCP, and hinged-cancellous bone at 2 weeks and 6 years were determined. The intraclass correlation coefficients of the inter-observer measurements for the first and second rounds were 0.67–0.94 and 0.79–0.96, respectively. The intraclass correlation coefficients of the intra-observer measurements for the first and second investigators were 0.89–0.96 and 0.66–0.94, respectively.

## Discussion

The most important finding of this study was the quantitative evaluation of the β-TCP resorption and bone formation in opening HTO. There are several reports that evaluate β-TCP resorption in opening HTO [1, 4, 11, 16, 19]. van Hemert et al. [19] developed a radiological rating system to monitor bone healing in opening wedge osteotomies. Remodelling of bone gaps filled with β-TCP granules or blocks with 70 % porosity was graded into five categories. The results were only analysed by conventional anteroposterior and lateral radiographs, potentially leading to observer variance of radiological measurements. Thus, a specific radiographic rating system is needed. As yet, no radiological rating system using CT images to monitor bone healing in opening HTO is available. CT values of 60 % and 75 % porosity β-TCP blocks at the centre of CT images at 2 weeks and 6 years were originally measured; however, CT images at 6 years showed that β-TCP with 60 % porosity was not uniformly resorbed (Fig. 1). This strongly suggested that measurements focused on a certain area (region of interest) of β-TCP were inadequate to evaluate β-TCP resorption. Thus, monitoring of the whole β-TCP implanted area was necessary. However, it was difficult to analyse CT values of the whole area. We therefore used the imaging software, Osirix, which was developed in 2004 by a team of radiologists from Geneva and UCLA, working on Apple computers, and can be downloaded free of charge. Use of Osirix allowed us to scan the whole area and measure CT values. β-TCP blocks were still visible at 6 years in some cases, but in most of the cases, it was hard to define the β-TCP implanted area. In those cases, we referred to CT images taken at 2 weeks to define the β-TCP-implanted area. The present study showed that there was no statistical difference between the CT values of the area implanted with β-TCP of 75 % porosity at 6 years and cancellous bone, indicating that β-TCP with 75 % porosity was completely resorbed and replaced by bone. In contrast, from the CT values, approximately 1/3 of β-TCP with 60 % porosity remained even 6 years after surgery. Figure 1 showed that CT values in the area with insufficient β-TCP implantation was lower than cancellous bone, indicating that poor bone formation occurred in this area. Thus, our evaluation system also proved to be successful at monitoring bone formation.

Meidinger et al. [15] reported on smoking as a risk factor of non-union in opening HTO, but the present study did not show significant difference of β-TCP resorption in the two patients who smoked.

Some limitations to this study are as follows. First, the CT values of each circumferential cortical bone of the tibia were excluded from each value of β-TCP and cancellous bone, because it was hard to distinguish newly formed bone from residual β-TCP. Second, it was hard to obtain completely matched images at 2 weeks and 6 years, even though we tried to measure the centre of the osteotomy plane. Third, the CT images were divided into three areas by tracing manually on the screen of the computer. Although this manual process caused inter- and intra-observer differences, intraclass correlation coefficients analysis showed substantial to almost perfect agreement between the two independent investigators.

The present study showed that β-TCP with 75 % porosity was completely resorbed and replaced by bone, but β-TCP with 60 % porosity was only partially resorbed. However, the amount of residual β-TCP with 60 % porosity was so small that it would not hinder total knee arthroplasty should it be necessary in the future. To promote complete remodeling of β-TCP, additional stimulations such as growth factors may also be necessary [13].

This system is the first to quantitatively evaluate the residual amount of β-TCP and bone formation in opening HTO. It will be a useful technique to evaluate β-TCP resorption and bone formation in any β-TCP implanted area.

## Conclusions

The aim of this study was to establish a novel evaluation system to monitor bone formation and β-TCP resorption in opening HTO using the image software, Osirix. The results showed that β-TCP with 75 % porosity was completely resorbed and replaced by bone, but β-TCP with 60 % porosity was only partially resorbed even 6 years after surgery. The imaging software, Osirix, enabled us to evaluate β-TCP resorption and bone formation in both opening HTO and other β-TCP implanted areas.
